# Total Wrist Arthroplasty—A Systematic Review of the Outcome, and an Introduction of FreeMove—An Approach to Improve TWA

**DOI:** 10.3390/life12030411

**Published:** 2022-03-11

**Authors:** Jörg Eschweiler, Jianzhang Li, Valentin Quack, Björn Rath, Alice Baroncini, Frank Hildebrand, Filippo Migliorini

**Affiliations:** 1 Department of Orthopaedics, Trauma and Reconstructive Surgery, RWTH Aachen University Hospital, 52074 Aachen, Germany; jli@ukaachen.de (J.L.); vquack@ukaachen.de (V.Q.); fhildebrand@ukaachen.de (F.H.); fmigliorini@ukaachen.de (F.M.); 2 Department of Orthopaedic Surgery, Klinikum Wels-Grieskirchen, 4710 Wels, Austria; bjoern.rath@klinikum-wegr.at; 3 Department of Orthopaedic and Trauma Surgery, Eifelklinik St. Brigida, 52152 Simmerath, Germany; alice.baroncini@gmail.com

**Keywords:** total wrist arthroplasty, TWA, FreeMove, systematic review

## Abstract

The Swanson silicone prosthesis was one of the first devices to realize total wrist arthroplasty (TWA). It has been used regularly since the early 1960s. This systematic review of the literature evaluated the status quos of TWA. The present study was conducted according to the PRISMA guidelines. A literature search was made in Medline, PubMed, Google Scholar, and the Cochrane Library databases. The focus of the present study was on implant survivorship and related functional outcomes. Data from 2286 TWA (53 studies) were collected. Fifteen studies were included for the analysis of implant survivorship. Fifteen studies were included for the analysis of pain. Twenty-eight studies were included for the analysis of the Disabilities of the Arm, Shoulder, and Hand (DASH) score. Grip strength was tracked in 16 studies. The range of motion (RoM) was evaluated in 46 studies. For supination and pronation, 18 articles were available. Despite some methodological heterogeneities, TWA may be effective and safe in pain reduction and improving function and motion. There is still a range for a future improvement of the procedure.

## 1. Introduction

Total wrist arthroplasty (TWA) is still a controversial issue but it has become a challenge to total- and sometimes also partial-wrist arthrodesis (TWAD/PWAD). Even today, TWA has not found widespread acceptance, and most surgeons prefer to recommend a TWAD to their patients [1]. For patients who present with advanced joint degeneration and painful wrist, TWA and TWAD/PWAD are appropriate options for reconstruction [2,3]. Especially TWA has been shown to be effective in improving quality of life in patients with wrist rheumatoid and osteoarthritis (RA/OA) [4,5,6,7]. In this case, conservative means have not provided adequate pain relief, and other motion-preserving procedures are impossible, hopeless, or have failed [3]. Patients eligible for TWA should report chronic pain (RA/OA, or posttraumatic arthritis), low-activity lifestyle, and the desire to preserve wrist motion and have adequate bone stock and good quality of the soft tissue [7,8]. Thus, TWA has been considered an option only for certain individuals with specific needs and desires for motion who clearly understand the risks and benefits (Adams, 2001).

Themistocles Gluck firstly performed the first TWA in 1891 (an ivory ball-and-socket device) [9]. The evolution of wrist implants has been slower than that of, e.g., hip, knee, and spine [10]. The lower prevalence of symptomatic wrist RA/OA and the use of other treatments, such as TWAD/PWAD, dampened the interest in the development of wrist implants [10]. Furthermore, the small size and complexity of the wrist joint are obstacles to engineering and manufacture [10].

During the 1960s, Swanson implanted a silicone spacer that could offer immediate stability for the radiocarpal joint [11]. Niebauer [12] added a foundation that allows the ingrowth of fibrous tissue without inhibiting motion. The second implant generation, introduced in the 1970s, was hard-bearing multicomponent prostheses. There is no consensus about the definition of second-generation implants [3,5,13]. Generally, these implants consist of a radial component and a carpal component that is fixed into one or more metacarpal bones after bone resection [3,5,14,15]. The third generation of TWA was characterized by moderate bone resection and avoided fixation in the metacarpal bones, except for an optional, short length of screw fixation in the index finger metacarpal [3,15]. Pyrocarbon was recently introduced as a single-component interposition arthroplasty [16,17,18] or hemiarthroplasty [19,20,21]. The fourth generation of TWA implants required screw fixation to the carpus, with a porous surface to increase osseointegration for uncemented implants [14,22,23,24]. Contrarily to previous generations, these implants could be implanted without cement [8,22,25].

The concept of TWA over traditional TWAD has gained popularity because recent developments in prosthetic design and intervention techniques have provided improvements in the functional performance and durability of TWA, leading to renewed interest, especially for RA patients [6,10]. Despite the popularity of TWA, the mid-term to long-term implant durability remains unclear [7]. A recent meta-analysis of 500 wrist replacements (18 studies) compared with 800 wrist fusions (20 studies) of Cavaliere and Chung [2] suggested that fusion provided equally good results and was, therefore, more cost-effective [13].

Because of the fact, that the results of TWA in terms of prosthesis survival have generally been poor compared to most other prostheses [26], the purpose of this study was to elongate the knowledge about TWA, doing a systematical review about the available evidence and to compare clinical and surgical outcomes among patients undergoing a TWA. The objective was to systematically analyze the literature concerning TWA using first-, second-, third-, and fourth-generation implants. The intention was to fulfil a comprehensive insight about the current performance of existing wrist implants. Furthermore, a new concept of TWA prosthesis called “FreeMove” and the idea behind it is briefly introduced.

## 2. Material and Method

Before the beginning of the systematic review, a protocol was defined outlining the search strategy, inclusion and exclusion criteria, and outcomes of interest. The present systematic review was conducted following the standard methodology outlined in the Cochrane Handbook [27], and the Preferred Reporting Items for Systematic Reviews and Meta-Analyses (PRISMA) statement guidelines [28,29].

### 2.1. Search Strategy

A literature search of Medline, PubMed, Google Scholar, reference lists, and the Cochrane Library databases was conducted. We used exploded MeSH terms and keywords to generate sets for the following themes: “Total Wrist Arthroplasty” and “Total Wrist Replacement” (TWR), “duration”, “wrist arthroplasty”, and “Total wrist arthrodesis”. We then used the Boolean term “AND” to find their intersection. Our search was unrestricted focusing primarily on the 2000 to 2021 period. After that, a second by scanning the reference lists of the papers first included was performed. No limits were used, including no language limits. Additionally, this basic approach was modified as necessary to search each electronic database. Furthermore, we contacted subject-matter experts in the field of TWA.

### 2.2. Inclusion Criteria

The general inclusion criteria were papers about TWA and TWR with clinical data on first-, second-, third-, and fourth-generation implants. Published studies were included in the analysis if (1) the design was a comparative study, (2) patients underwent primary TWA/TWR, (3) and at least one quantifiable pre-specified outcome measure was reported.

### 2.3. Exclusion Criteria

We excluded papers about cadaveric studies, biomechanical studies, studies not accessible in journals, and books or online reviews without primary data. Double publications and articles with an overlap of cases were relative exclusion criteria. Articles not written in English or German were evaluated based on an English abstract, if available.

### 2.4. Study Reviews

Two reviewers (JE and FM) independently analyzed the resulting articles and conducted an initial review for eligibility based on title and abstract. Studies that were not related to our research question were immediately excluded. The remaining studies were then divided among the two reviewers such that both reviewers independently assessed each to confirm final eligibility. We developed and piloted a standardized form for collecting data related to study methodology, participant characteristics, and outcomes of interest. Data extraction was independently performed by both reviewers. For the statistical analysis, the tools of MS-Excel (Microsoft, Office package 2016) were used.

### 2.5. Quality Assessment and Handling of Data

The focus was on, e.g., the number of cases, the duration of TWA, and the observation period. TWA duration was evaluated based on papers mentioning the keyword implant survival without any restriction. The function was evaluated by validated and relevant outcome measurement tools such as the Disabilities of Arm, Shoulder, and Hand (DASH/QuickDASH), or the worst pain reported by a Visual Analog Score (VAS).

### 2.6. General Demographic Data

Table 1 is a summary of the overall patient demographics. The majority of the data are based on RA cases. Additionally, diagnoses are increasingly represented in recent publications.

### 2.7. Statistical Analysis

Summary statistics including mean values were calculated. Studies in this systematic review include partly small case series with nonrandomized design and are largely retrospective. This level of evidence contains inherent biases, making statistical testing inappropriate [34].

Therefore, mean values were calculated to highlight general trends. The limitation is here, that a conclusion whether statistically significant differences exist cannot be reached.

## 3. Results

### 3.1. Study Selection

**Figure 1:**
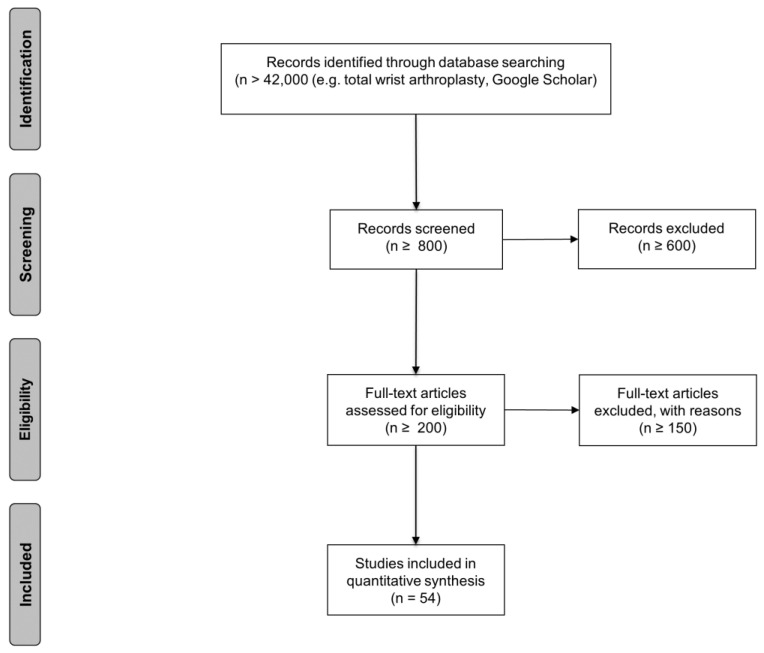
Study selection flow diagram of the systematic literature search for TWA. A total of 54 articles were included for a qualitative evaluation of the clinical outcome (n = numbers of papers). Inclusion and exclusion criteria were determined before the literature search. Studies from the literature search that were excluded through title and abstract review were studies of wrist arthrodesis, proximal row carpectomy, and fusion interventions arthroplasty.

The Figure 1 shows the study selection flow diagram of the systematic literature search for TWA.

### 3.2. Selected Publications

More than 42,000 papers were eligible as the outcome of the literature search (Figure 1). The screening of the publication lead to an exclusion of more than 600 articles. We checked the full text of round about 200 papers, which lead us to 54 studies with an input for analyzation. We found four systematic reviews about TWA [5,22,34,35].

The eligible studies represent a maximum of 2286 cases (Table 1).

### 3.3. Included Prosthesis Models

The Table 2 gives an overview about the included types of prostheses.

### 3.4. Primary Outcome—Duration of Implants

Table 3 gives an overview about the duration of the included different prosthesis models.

The Kaplan–Meier approach (Table 3) is one of the best options to measure the fraction of subjects (in our case the duration of the implant) living for a certain amount of time after treatment. 

In clinical investigations, the effect of a therapy is assessed by measuring the number of subjects that survived after that therapy over a period of time. The time starting from a defined point to the occurrence of a given event, e.g., the revision of the implant is called survival time and the analysis of group data is called survival analysis.

The life span (Table 4) means the period of time between the implantation of the prosthesis and the failure (revision) of it.

### 3.5. Secondary Outcome—Patient-Reported Measures of Pain

Pain is a critical outcome because it is the symptom that most often leads patients to seek surgical intervention [34]. Reporting was more complete for postoperative pain than for preoperative pain. It was still limited by inconsistent measures.

The Visual Analogue Scale (VAS) (Table 5) is a measurement instrument that tries to measure a characteristic or attitude that is believed to range across a continuum of values and cannot easily be directly measured [78,79]. In case of epidemiologic and clinical research, the VAS is used to measure the intensity or frequency of various symptoms [78,80].

The DASH Score (Table 6) is a questionnaire for orthopedic patients and was developed in 1996 by the Council of Musculoskeletal Specialty Societies, the American Academy of Orthopaedic Surgeons, and the Institute for Work and Health Canada. The DASH score was designed to be a standardized assessment of the impact on the function of a variety of musculoskeletal diseases and injuries in the upper extremity [81].

The DASH score comes along with a comparable responsiveness compared to other joint and disease-specific measures. It ranks from preoperatively 91 (highest) to 38 (lowest), and in the postoperative situation from 60.7 (highest) to 11.4 (lowest). It comes along with a large range.

### 3.6. Secondary Outcome—Patient-Reported Measures of Function

The grip strength (Table 7) is the force applied by the hand to pull on or suspend from objects. It can be assessed through standard methods and is a specific part of hand strength.

Grip strength is a general term also used to refer to the physical strength of a patient, to the muscular power and force that can be generated with the hands. This parameter depends on the physical condition of the patients.

The Table 8 and Table 9 show the Range of Motion (RoM) of the wrist and the pro- and supination of the forearm.

## 4. FreeMove—A New Approach for TWA

### 4.1. Introducing the Concept of FreeMove

**Figure 2:**
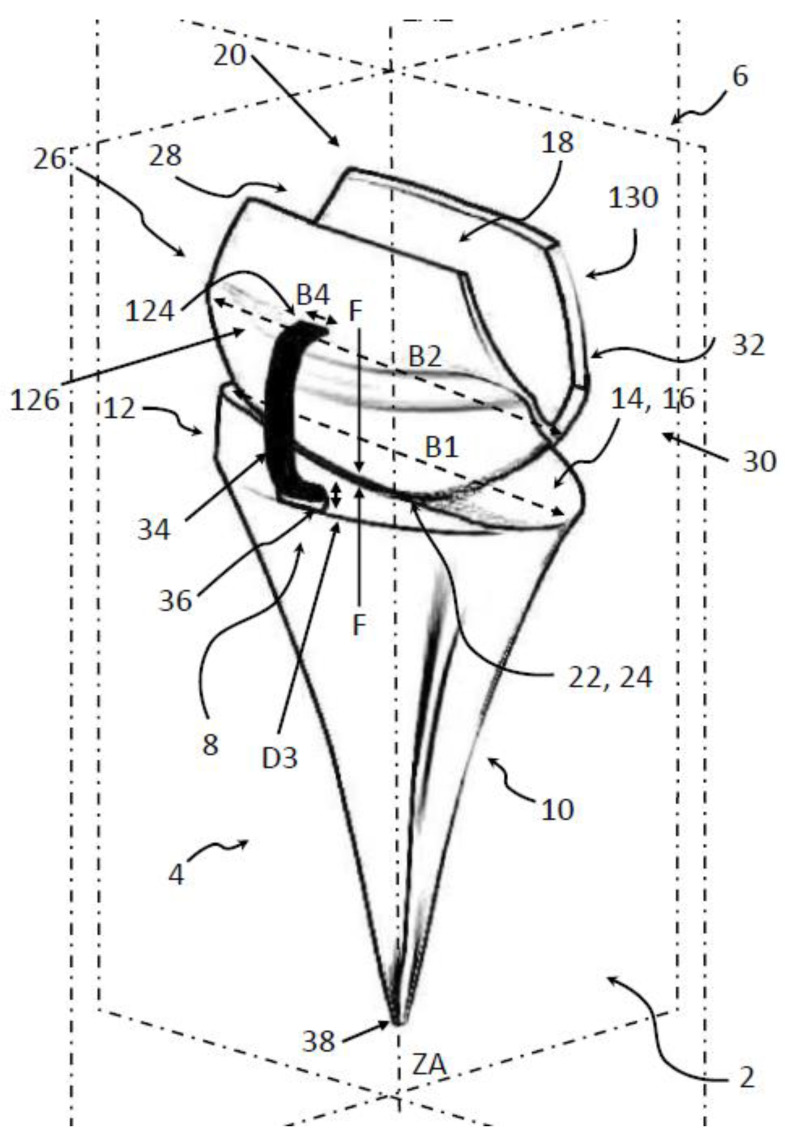
FreeMove prosthesis with different features: 8: luxation protection; 6: carpal component, 4: radial component (for a full description, see [88]).

Early generations of implants had high complication and failure rates [84]. The common modes of failure have been fracturing, loosening, pain on pronation and supination at the level of the distal radioulnar joint, and muscle soft-tissue imbalance. Problems coming along with for example distal component loosening and wrist imbalance with existing prostheses were the impetuses for developing, e.g., the Universal total wrist implant [85]. Total wrist arthrodesis for the salvage of failed TWA results in a complete limitation of wrist FE and RUD. It was suggested that attempts to recreate the natural joint should be avoided, and different materials and methods for fixation should be considered for new implants [86,87]. To prevent these limitations, the new approach FreeMove was developed (see Figure 2). We developed the new wrist prosthesis from 2018 to 2021.

Our intention of the new approach is that the implantation requiring only minimal bony resection, an uncemented (optional a cemented) radial component, firm and reliable cemented distal fixation via covering the proximal carpal row bones, and a prosthesis that involves simple instrumentation.

### 4.2. The Principal Idea of FreeMove

The design of wrist prostheses has evolved based on clinical experience and kinematic and biomechanical studies [85]. This new implant design of FreeMove differs from the reported total wrist prostheses (Table 2) by transforming the wrist to an ellipsoid joint with a polyether−ether−ketone (PEEK) bearing and a variable center of articulation. The ellipsoidal design was found to accommodate greater width of the concave proximal component, resulting in better capture and prosthetic stability [85]. The articulating surfaces of the carpal and radial components create a dual-axis articulation that is best suited for radial and ulnar motions [85].

We try to use PEEK because wear, metallosis, and the systemic influence of metallic ions were suspected problems. Press-fit fixation on the radial part secures primary stability. The distal part covers the proximal carpal row, and modularity on both sides of the joint simplifies the replacement. Furthermore, the intention of using PEEK is here to reduce wear and the need to remove the bone. If the prosthesis fails, a second TWA prosthesis or a wrist arthrodesis should be easy because so little bone needs to be removed. With this approach, the current fixation technique of the distal part of the wrist prosthesis with a screw in the, e.g., third metacarpal bone will be avoided. This decreases the risk of screw loosening may eventually also decrease the risk of loosen other prosthesis parts by enabling a more physiological movement of the implant.

The design included a PEEK-on-PEEK coupling with an ovoid surface interaction with more or less an elliptical articulation. The elliptical concept has been stable and resulted in a good range of motion. Furthermore, to avoid luxation, protection was built in via an artificial ligament. This should improve the stability of the joint. The radial component includes an inclination of 20° to mimic the physiological orientation of the articular surface of the normal distal radius [85].

In the normal wrist, the center of rotation for FE and RUD should be located in the head of the capitate, which is slightly distal to the center of the prosthesis [89,90,91]. The introduced prosthesis has no fixed center of rotation. The distal part can slide and rotate on the proximal (radial) part depending on the external load.

The manufacturing of the new prosthesis is addressed via 3D-printing. This allows a patient-specific design and adaption, respectively. From a CT-scan, the geometry of the wrist could be reconstructed and transferred to an individual prosthesis design. The including of a luxation protection via a surrounding robe increases the function of the implant and was to our best knowledge never introduced before.

### 4.3. Conclusion and Future Work Concerning FreeMove

This new implant differs from most of the reported total wrist prostheses by transforming the wrist into an ellipsoid floating joint with a PEEK-on-PEEK bearing and a flexible center of articulation. Considering the fact that the wrist joint articulates with six other bones (radius, ulna, capitate, trapezoid, trapezium, and hamate) and shows rotational and also translational motion, our impression is that any wrist prosthesis must replicate more or less patient-specifically the original shape of the joint surface as precisely as possible to minimize non-physiological kinematics and wear. This requires a patient-specific adapted implant. Future steps are planned with several experiments with this concept carried out on cadaver wrists.

## 5. Discussion

The wrist was one of the first joints treated by a prosthesis. Given the lower prevalence of symptomatic wrist OA/RA and the ease and predictability of TWAD, the evolution of TWA has lagged behind advancements made in large joint replacements [64,92]. The main potential advantage of TWA over TWAD is the potential for preservation of movement for patients with painful wrist OA/RA. This study adds to the current evidence in support of the use of TWA in all kinds of patients and kinds of the prosthesis. This overview should allow obtaining an impression of the performance of TWA. However, the limited available data limited the current spread of such implants, and future studies are required to overcome current limitations.

First experiences with TWA wrist are based on developments by Meuli and Volz [93,94]. Early outcomes showed a high rate of complications at an early stage with malpositioning, dislocation, and loosening of the components [93,94]. In their original form, they are no longer implanted [94]. Because of the complex intervention and the semi-optimal results, TWA is not a routine process. The majority of the data are based on rheumatoid cases (59.5%), although other diagnoses are increasingly represented in recent publications.

The strength and advantage of the presented systematic review is the comprehensive literature search and the assessment of the methodological quality of the available data.

### 5.1. Duration

Based on the currently available evidence comparing outcomes following TWA/TWR, we cannot conclude the superiority of the success of such an intervention. Articles provided Kaplan–Meier survivorship curves are shown in Table 3, and one paper provided the life span of the implants (Table 4).

There was a wide variation in survival from 42% [31] for the Swanson silicone prosthesis to 57% after five years [26] for the Elos prosthesis, to 94% after 10 years [32] for the Remotion prosthesis, as shown in Table 3. The Elos prosthesis displayed a very steep failure rate on the Kaplan–Meier curve over the first 4 years before reaching a plateau [22,26].

The UWP-1 showed a survival rate of 60% after 7 years [68], to 91% after 7.8 years [52], and to 75% after 15 years [65]). The Remotion prosthesis showed different rates of survivorship starting from 99% after 5 years [33], to 94% after 8 years [33], to 94% after 10 years [32]. The Biaxial prosthesis showed rates from 81% after 7 years [43] to 78% after 12 years [33]. The UWP-2 was rated with a survivorship of 83% after 10 years [32]. The Maestro prostheses showed a rate from 95% after 8 years to 93% after 10 years [32]. Some articles provided Kaplan–Meier survivorship curves, with censored data representing those lost to follow-up, including deaths [22,26,43,58,68].

Because of the heterogeneous studies, it could not be decided which implant is the best. In the end, the conclusion could be that an improvement of the existing procedure of TWA including the current used implants must be one future goal. In comparison to the success of total hip and total knee arthroplasty, TWA has to be considered for further research.

### 5.2. Pain

Pain is a complex and patient-specific experience, and attempts to make valid assessments of it have been fraught with difficulties. Pain is influenced by different factors and depends on the personal constitution of the individual patient. Fifteen articles detected the pain. The mean value preoperatively was 7.5, and the postoperative mean value was 2. A decrease in pain could be seen and thus an increase the quality of life for the patients. The problem in the case of pain as a valid parameter to benchmark the intervention outcome is the subjectivity. Patients handle the situation in case of pain more or less individually. The outcome depends on the individual sensation of each patient.

### 5.3. Disabilities of the Arm, Shoulder, and Hand (DASH)

Functional scores as measured by DASH appear to improve at follow-up post-TWA. The DASH score is one of the most established questionnaires for disorders of the upper limb. The collection and analysis of the results are easy to use and interpret. The mean value for the preoperatively DASH score was 58, and for the postoperative situation, it was 36. There was, in the mean, an increase in the DASH score. That shows that the approach supporting the damaged wrist joint with an artificial implant leads to an increase in the quality of life for the patients. In consideration of the duration of the included implants, to date, it is only a temporary solution with a high risk of revision interventions.

### 5.4. Grip Strength

It is difficult to objectively quantify grip strength improvement. The reason for that was inconsistent in the pre- and postoperative measurements. Additionally, the varying means of measurement and different acquisition methods lead to confusion. We focused on articles that acquired the grip strength in kg. The mean value for the preoperative grip strength was 12 kg, and for the postoperative situation, it was 18 kg. There was, in the mean, an increase in the grip strength. While grip strength alone does not predict the performance of patients’ outcomes, periodic measurement of grip strength could be beneficial in terms of patient performance and injury prevention. Only mirroring the postoperative situation does not show the future development of the patient situation. Additionally, the influence of grip strength as a parameter of success is not clear. In Table 7, the grip strength in case of the preoperative status shows the diversity of this parameter: the lowest grip strength was 2.1 kg up to 21 kg, and in the postoperative situation, it starts at 7.9 kg and goes up to 32 kg. This shows a large range of this parameter. A correlation with a body/trainings condition of the patient must be considered to judge the measurement results. The establishment of a baseline data in the context of grip strength would be a valuable approach to rate therapy outcomes.

### 5.5. Range of Motion

The results for the RoM suggest that, with TWA, the postoperative is preserved compared with preoperative RoM. There exists a functional range of wrist motion (based on activities of daily living) that has been defined as 5° of flexion, 30° of extension, 10° of radial deviation, and 15° of ulnar deviation [95,96,97,98].

Of the included articles in our study, 46 papers analyzed the RoM. There is a mean RoM postoperatively for the flexion of 32°, for the extension of 31°, with a mean overall flexion−extension of 63°. Furthermore, there exists a mean RoM for the radial deviation of 9°, for the ulnar deviation of 10°, with a mean overall radial−ulnar deviation of 28°. When the mean RoM of the postoperative situation is compared to the functional range of wrist motion, flexion, extension, and ulnar motion fit well. Only the radial motion is too small.

Of the included articles in our study, 18 papers additionally analyzed the range of supination and pronation. There is a mean range postoperatively for the pronation of 72°, for the supination of 72°, with a mean overall motion of 155°. There is an improvement compared to the preoperative situation where the pronation was 67° and the supination was 61°, with a mean overall motion of 137°. When the mean range of the postoperative situation is compared to the range preoperatively, the motion increased nearly about 20°.

Not all papers compared the preoperative with the postoperative situation. Some articles provided only a range in the data, and others expressed this in detail split to the single motion. There is a wide RoM presented by all studies, with a wide spread of data. The question is how valuable this parameter is to obtain an impression and how good the outcome of the therapy is.

### 5.6. Limitations in General

Any review of the literature is limited by the quality of published reports. The presented study is limited by the inability to perform a quantifiable meta-analysis in the case of analyzing patient-reported pain and function because of missing randomized clinical trials of TWA compared to TWAD. Moreover, given the variability of outcome measures, detailed pros and cons of such intervention were not possible to discuss. The available evidence is limited, and the current literature surely benefits from further biomechanical and clinical investigations.

Given the limited number of papers analyzing TWA, we decided against establishing an exclusion cut-off based on study design and eliminating potentially useful data from our review. This led to the inclusion of some studies of poor methodological rigor that likely represent bias.

Standard statistical testing requires input of high-quality data obtained through standardized methods and detailed reporting of all outcomes. Our statistical analysis was limited to calculation of mean values, which provide a summary estimate of the results.

Furthermore, the inclusion of complication rates, revision rates, Patient-Rated Wrist Evaluation (PRWE), the explicit results for each prosthesis model, the explicit results for each pathology, satisfaction, and radiological output was too much for this paper, and it is planned to realize this in an additional publication.

### 5.7. Methodological Quality of Included Studies

The included studies sometimes demonstrate moderate methodological quality and a likelihood of (systematic) error. There is an inaccuracy, e.g., in describing the included patients vs. procedures, describing exactly the numbers of complications, and the numbers of analyzed procedures at each time point. Sometimes it was difficult to find out the correct numbers for these parameters.

## 6. Conclusions

Despite advances in the field of arthroplasty, TWA significantly lags behind, e.g., total knee or hip arthroplasty. Besides this fact, some general conclusions are possible: it seems that TWA has a strong potential for improvement of function through pain reduction and preservation of mobility [5]. It seems also that TWA is a possible alternative to total wrist arthrodesis in patients with painful, debilitating degenerative pathologies of the wrist [92].

The multiple numbers of implants with varying designs indicate a lack of universal acceptance for wrist anatomy and biomechanics.

There is a need for additional research. The focus should be on long-term results achieved through large retro-/prospective studies. Furthermore, the initiation of a surveillance register of implants should be a next step that is not available to date [5]. This investigation emphasizes the need for methodologically rigorous, multi-centered, prospective, randomized controlled trials with predefined reporting, standardized follow-up intervals, outcome measures, anesthesia and rehabilitation protocols, and reporting of pre-operative indication [5]. In reviewing the different designs of the prostheses and the recent outcomes of the different implants, only time will tell if these implants will further the advances in TWA [92].

Furthermore, the question as to which causes and consequences of the periprosthetic loosening must be exposed by multiple methods to improve the outcome [5]. Another improvement for a better comparison of TWA outcome could be better standardization of data acquisition and investigation methods of the different parameters for benchmarking the TWA results.

## Figures and Tables

**Table 1:** Patient demographics—overview (right side number in brackets: references).

Total Number of Procedures/Prosthesis	2286
Number of different prostheses	20
Mean follow-up ranges from (month)	11 [17]
Mean follow-up ranges to (month)	213.6 [30]
Average age ranges from (years)	47 [31]
Average age ranges to (years)	68.3 [30]
Youngest patient (years)	17 [26]
Oldest patient (years)	88 [32,33]
Male:Female (ratio)	65.5%:34.5%
Rheumatoid arthritis (%)	59.5%

**Table 2:** Short description of the included prosthesis models of the 54 references. The table gives an overview about technical parameters of each of the included wrist prostheses. It shows the prosthesis type, the manufacturer, and a short description of each prosthesis.

Prosthesis	Manufacturer	Short Description
**Biaxial prosthesis** [26,32,36,37,38,39,40,41,42,43]	DePuy, Warsaw, IN, USA	The Biaxial prosthesis: is a 3-component prosthesis, composed of a metacarpal (distal) and a radial (proximal) component, and the stems have porous-coated surfaces. includes an ultrahigh molecular weight (UHMW) polyethylene sliding core. has a rounded, unconstrained, articulating interface, oriented in the plane of wrist movement. includes a distal component that consists of a larger stem for insertion into the third metacarpal and a small stud for insertion into the trapezoid to stabilize it during rotation.
**Elos prosthesis** [26]	Swemac, Linkoping, Sweden	The Elos prosthesis: with its different versions were all preliminary types of the Gibbon prosthesis. version 1 had a short metacarpal screw that was fully threaded, as was the radial screw. in later versions, the metacarpal screws were longer, the diameter smaller, and the heads lower.
**Gibbon prosthesis** [26]	Swemac, Linkoping, Sweden	The Gibbon prosthesis: is a modular (4-component) prosthesis. articulation is cobalt chrome-molybdenum alloy treated with chromium nitride stem is made of titanium alloy blasted and coated with a resorbable calcium phosphate combination. was CE-marked in late 2005 and changed the name to Motec in 2010, without any change to the prosthesis.
**Motec prosthesis** [44,45,46]	Swemac, Linköping, Sweden	The Motec prosthesis: is a cementless modular metal-on-metal ball-and-socket prosthesis. includes grit-blasted surfaces of the screws which were coated with resorbable calcium phosphate. comes along with three lengths of radius component (32, 38, and 44 mm) and of capitate/third metacarpal component (45, 50, 55, 60, and 65 mm) screws, the latter in two thicknesses. has three neck lengths for tension adjustment.
**Destot implant** [47]		The Destot implant: is a non-constrained, metal-polyethylene condylar prosthesis. has carpal components made of 316 L steel. stems have a sandblasted/porous-coated surface to eliminate the need for cement and to enhance osseointegration. has a concave articular surface of the radial component, which is made of UHMW polyethylene. The stem of the radial component is V-shaped and has grooves at either side for bone growth.
**Meuli Wrist Prosthesis** **(third revised implant)** [41,48,49]		The prosthesis MWP III (Meuli Wrist Prosthesis/third revised implant): is a titanium 6-aluminum 7-niobium wrought alloy Protasul 100. The surface is corundum rough blasted. The ball head is coated with titanium nitride. The cup inset is made of UHMW polyethylene. The special design of the prosthesis with two sizes in right- and left-hand versions helps to center and balance it. is designed so that it could be cemented or uncemented.
**Anatomic physiologic wrist prosthesis** **(APH)** [50]	Implant-Service Vertriebs-GmbH, Hamburg, Germany	The Anatomic physiologic wrist prosthesis is an uncemented cobalt–chrome prosthesis, combining titanium/titanium articular surfaces a hydroxyapatite-coated cobalt–chrome prosthesis with a titanium coating of the articular surfaces. The radial component has an articular surface inclination of 10° toward the ulna. The carpal component is anchored with its tip in the third metacarpal bone and the distal carpal bones. It has a mobile bearing surface with a radial inclination of 10°. The radial component is made in four sizes, and the carpal component is available in one standard size.
**RWS Prosthesis** [51]	HowmedicaTM, Pfizer Hospital Products Group, The Netherlands	The RWS Prosthesis is a semi-constrained device that has three components: a radial component consisting of a UHMW polyethylene insert in a Vitallium tray and a metacarpal component. The design allows for a mechanical arc of 100° motion in the anteroposterior plane, 40° of radio-ulnar deviation, and minimal axial rotation. The center of rotation is located at the proximal pole of the capitate and is placed slightly palmar and ulnar to the long axis of the radius by off-setting the intra-medullar system of the radial component. Carpal height can be restored by choosing variable thickness UHMW polyethylene insert components.
**Universal-prosthesis** **(second generation) (UWP-2)** [32,33,41,52,53,54,55,56]	Integra Life Sciences, Plainsboro, NJ, USA; (previously manufactured by Kinetikos Medical Inc.)	The Universal 2 prosthesis is a modified Menon (Universal) prosthesis. It is an unconstrained joint with a cobalt chrome radial component and titanium carpal component, each with a beaded porous coating for osseous integration. The ellipsoidal design of the carpal component enables a more consistent contact area with the radial component throughout the range of motion, compared with the original toroidal shape. The increased radial component width provides greater capture of the carpal component, thus conferring greater rotational stability.
**RE-MOTION** [32,33,57,58,59,60,61,62]	Small Bone Innovations Inc; Morrisville, PA, USA	The RE-MOTION (formally AVANTA) TWR: is an uncemented implant, it includes screw fixation into the carpus, bone preserving, and deep radial articulation (prevent subluxation) and is designed as a mobile bearing ellipsoidal polyethylene component. Resection of bone is required upon prosthesis insertion, which preserves the ligamentous and soft tissue attachment of the wrist. is an elliptical ball and socket design of radial and carpal Cr-Co components that are titanium-coated, and an intercalated polyethylene component that mainly articulates with the radial component but also permits a rotational articulation of 20 degrees with the carpal plate. The carpal plate has fixated the carpus by its stem and two screws, of which only the most radial may penetrate the metacarpal for a very short distance even though many advocate not doing so aimed to be to the carpus and minimally in the metacarpals. The fixation is often performed without cement.
**Universal prosthesis, first-generation** [30,53,59,63,64,65,66,67,68]	Kinetikos Medical Inc.,4115 Sorrento ValleyBlvd., San Diego, CA, USA	The Universal Wrist Implant: is a non-constrained joint and is available in three sizes. radial and carpal components are made of titanium. has a concave articular surface of the radial component with 20° inclination similar to the articular surface of the radius. the stem of the radial component is Y-shaped and has tie mesh on either side for bony ingrowth. can be inserted with or without bone cement. the carpal component is ovoid and matches the cut surface of the carpal bones. has a convex high-density polyethylene insert that slides over the carpal plate.
**Maestro** [4,32,33,59]	Biomet, Warsaw, IN, USA	The Maestro prosthesis is designed to replace the distal end of the radius as part of a TWA to treat a severe bone fracture or degenerative disease. is made of titanium or cobalt–chrome and is implanted into radial bone proximally; interfaces with a polyethylene (PE) spacer distally. could be implanted with or without bone cement.
**Total modular wrist prosthesis** [69]	Micromed, Germany	The Total modular wrist prosthesis is available as a constrained or non-constrained device consisting of four components. comes with a titanium radial component that articulates with a titanium carpal plate with a variable thickness polyethylene insert in between. has separate shapes of the insert to provide a constrained or non-constrained version. the carpal plate is fixed to the second, third, and fourth metacarpal bones by titanium screws of variable length. comes along with an optional ulna component prosthesis consisting of a proximal screw and blunt tip at the distal end articulates with the radial component to form a ball-and-socket type joint. components are coated with hydroxyapatite and an uncoated radial component is available for cemented purposes.
**Modular Physiological Wrist prosthesis** (MPW) [70]	Link Company™, Hamburg, Germany	The Modular Physiological Wrist prosthesis: is a modularly designed, cementless, implantable Titanobium endoprosthesis. a special feature is the encapsulated sliding pairing of the distal olive, which is intended to imitate the mobility of the intercarpal joint line. has a solution for bad bone quality, and various components are available, including a coupled implant.
**Resurfacing Capitate Pyrocarbon Implant** **(RCPI)** [20,21]	Tornier, Grenoble, France	The Resurfacing Capitate Pyrocarbon Implant: contains a central core of graphite resurfaced with pyrocarbon. has good biochemical and biomechanical compatibility, excellent wear resistance, and an extremely low coefficient of friction. comes along with a modulus of elasticity of the material, which is comparable with that of the bone. is a single block, with a 15° tilt between the stem and head. is a cementless prosthesis. has commercially available head diameter sizes of 14 and 16 mm.
**Volz prosthesis** [71,72,73]	Stryker, Mahwah, NJ, USA Howmedica Company, Rutherford, NJ, USA	The Volz prosthesis: is a single/double-stemmed prosthesis is made of CoCr metacarpal and radial components includes a polyethylene articular component proximally
**Trispherical total wrist prosthesis** [74]		The trispherical total wrist prosthesis: consists of metacarpal and radial components articulated with a polyethylene bearing and an axle restraint. the metacarpal component has a central stem for the third metacarpal, with an offset stem for the base of the second metacarpal and scaphoid. the radial component has a stem for the radius and the articulation is offset ulnarward so that the instant center of the wrist is within the capitate. the radial component has a 12-degree palmar tilt. The high-density polyethylene bearing fits into the metacarpal component and forms a ball-and-socket joint with the radial sphere. is designed to provide 15 degrees of radial and ulnar deviation, 90 degrees of flexion, and 80 degrees of extension without constraint.
**Amandys** [16,17,18]	Tornier, Bioprofile	The Amandys implant: is a non-restrictive implant made of pyrocarbon termed Amandys. Pyrocarbon possesses excellent biocompatibility, an elasticity modulus close to that of bone tissue, and virtually does not wear out due to a very low friction coefficient against these structures, thus causing no wear to the bone. comes in eight sizes with two widths (24 and 26) and four different thicknesses (S, M, L, and XL). has an almond shape with two surfaces of different convexity, the most convex coming into contact with the radial projection and the other into contact with the capitate bone. is cementless, monoblock, and mushroom-shaped, with a central core of graphite (99 percent), covered by a thin layer of pyrocarbon (1 percent).
**Swanson wrist implant** **(Silicone implant)** [31,75,76]	Wright Medical, Memphis, TN, USA	The Swanson Wrist Joint Implant is a one-piece intramedullary stemmed implant fabricated from implant-grade silicone elastomer. is designed for use in implant resection arthroplasty of the radiocarpal joint. is available in five sizes to satisfy most anatomical requirements. has a wide mid-section to match the width of the radius. comes along with a shorter distal stem that extends through the carpus into the base of the third metacarpal.
**DARTS-Total Wrist System** [77]	Teijin Nakashima Medical Co., Ltd., Okayama, Japan	The DARTS—Total Wrist System: is a new semi-constrained total wrist prosthesis that positions the joint line at the midcarpal joint to limit stress on surrounding soft tissues. consists of UHMWPE radial and titanium-6 aluminum-4 vanadium (Ti-6Al-4V) carpal components, Ti-6Al-4V bone screws, and a cobalt-chromium-molybdenum (Co-Cr-Mo) carpal head. has a radial component with an offset volarly and radially. includes an articular surface of the carpal component, which forms an ovoid to reproduce the physiological movements of the wrist. The carpal component for the base of the third metacarpal bone has a volar flange that was added to resist the posterior and rotational displacement forces thought to contribute to early carpal loosening and is augmented by two cancellous screws placed in the second and fourth metacarpals. flexion–extension axis is rotated outwardly by 10 around the line of intersection of the horizontal plane and the distal articular surface of the radial component to provide wrist movement from radial-extension to ulnar-flexion. is available in three sizes; the appropriate size was determined using preoperative templating on radiographic images of the radius and metacarpals and the intraoperative findings.

**Table 3:** Duration of different prosthesis models selected out of the 54 references. The table shows the output concerning the duration of the wrist prostheses. The primary author, the publication year, and the Kaplan–Meier survivorship including the type of included prosthesis are listed (./.: Data are not available within a study).

No.	Reference	Year of Publication	Kaplan-Meier	Time-point	95% Confidence Intervals		Type of Implant
			[%]	[years]	Range from	Range to	
**1**	Jolly [31]	1992	42.0	7.0	./.	./.	Swanson
**2**	Cobb [36]	1996	83.0	*	72.0	93.0	Biaxial
**3**	Takwale [42]	2002	83.0	8.0	68.0	98.0	Biaxial
**4**	Levadoux [47]	2003	85.0	4.0	./.	./.	Destot
**5**	Kurkhaug [26]	2011	85.0	5.0	78.0	93.0	Biaxial
			57.0	5.0	33.0	81.0	Elos
			77.0	4.0	30.0	90.0	Gibbon
**6**	van Harlingen [43]	2011	81.0	7.0	64.0	91.0	Biaxial
**7**	Ward [68]	2011	75.0	5.0	./.	./.	UWP-1
			60.0	7.0	./.	./.	
**8**	Boeckstyns [58]	2013	90.0	6.0	./.	./.	Remotion
**9**	Sagerfors [33]	2015	84.0	5.0	./.	./.	Biaxial
			81.0	8.0	./.	./.	
			78.0	12.0	./.	./.	
			99.0	5.0	./.	./.	Remotion
			94.0	8.0	./.	./.	
			95.0	8.0	./.	./.	Maestro
**10**	Badge [52]	2016	91.0	7.8	84.0	91.0	UWP-1
**11**	Gil [65]	2017	78.0	15.0	62.0	91.0	UWP-1
**12**	Honecker [62]	2017	95.7	4.0	./.	./.	Remotion
			91.3	6.0	./.	./.	
			69.0	8.0	./.	./.	
			69.0	10.0	./.	./.	
**13**	Fischer [32]	2020	94.0	10.0	./.	./.	Remotion
			86.0	10.0	./.	./.	Biax
			83.0	10.0	./.	./.	UWP-2
			93.0	10.0	./.	./.	Maestro
**14**	Biehl [70]	2021	33.0	6.9	./.	./.	MPW

* last follow-up.

**Table 4:** Life span of BIAX and Volz prosthesis. One publication listed the life span of the included prosthesis type instead of the Kaplan–Meier survivorship.

Reference	Year of Publication	the Life Span of the TWA	Range from	Range to	Type of Implant
		[Month]	[Month]	[Month]	
Ekroth [30]	2012	93.6	36.0	132,0	UWP-1

**Table 5:** Measurement of pain (VAS 0–10)—the table shows the reference, the year of publication, and the number of included patients/prostheses for the follow up investigation. (./.: Data are not available within a study).

No.	Reference	Year of Publication	Number Included for Follow Up	Worst Pain Reported by Visual Analog Score (VAS) (0–10)							
				Preoperatively (n)	Mean	Range from	Range to	Postoperatively (n)	Mean	Range from	Range to
**1**	Kistler [75]	2005	27.0	27.0	6.5	./.	./.	27.0	1.8	./.	./.
**2**	Bidawi [57]	2012	10.0	10.0	8.5	./.	./.	10.0	3.2	./.	./.
**3**	Cooney [59]	2012	39.0	39.0	7.0	./.	./.	39.0	2.3	./.	./.
**4**	Ekroth [30]	2012	12.0	12.0	./.	./.	./.	7.0	0.3	./.	./.
**5**	Nydick [4]	2012	23.0	23.0	8.0	./.	./.	23.0	2.0	./.	./.
**6**	Badge [52]	2016	85.0	47.0	8.1	3.0	10.0	61.0	5.4	0.0	10.0
**7**	Chevrollier [63]	2016	15.0	15.0	./.	./.	./.	15.0	2.0	0.0	7.0
**8**	Gil [65]	2017	39.0	39.0	8.6	./.	./.	39.0	0.4	./.	./.
**9**	Honecker [62]	2017	23.0	23.0	6.8	./.	./.	23.0	2.8	./.	./.
**10**	Pfanner [55]	2017	23.0	23.0	9.0	./.	./.	23.0	0.8	./.	./.
**11**	Giacalone [20]	2017	25.0	25.0	./.	./.	./.	25.0	2.0	./.	./.
**12**	Bellemere [18]	2019	51.0	51.0	6.5	./.	./.	51.0	2.3	./.	./.
**13**	Froschauer [60]	2019	39.0	39.0	7.0	./.	./.	39.0	2.0	./.	./.
**14**	Biehl [70]	2021	34.0	34.0	7.0	./.	./.	34.0	1.8	./.	./.
**15**	Lestienne [16]	2021	28.0	28.0	6.0	1.0	8.0	28.0	2.0	0.0	7.0

**Table 6:** DASH (0-100) (./.: Data are not available within a study).

No.	Reference	Year of Publication	Number of Procedures Included	Number Included for Follow Up	Disabilities of the Arm, Shoulder, and Hand (DASH) (0–100)							
			(n)	(n)	Preoperatively (n)	Mean	Range from	Range to	Postoperatively (n)	Mean	Range from	Range to
**1**	**Divelbiss** [64]	2002	8.0	8.0	8.0	./.	./.	./.	8.0	22.4	./.	./.
**2**	Strunk [41]	2009	34.0	34.0	./.	./.	./.	./.	34.0	60.8	20.0	97.5
**3**	van Winterswijk [67]	2010	17.0	17.0	17.0	91.0	./.	./.	17.0	65.0	./.	./.
**4**	Ward [68]	2011	20.0	20.0	10.0	62.0	42.0	80.0	10.0	40.0	18.0	80.0
**5**	van Harlingen [43]	2011	32.0	32.0	31.0	66.0	./.	./.	31.0	34.0	./.	./.
**6**	Cooney [59]	2012	46.0	30.0	./.	./.	./.	./.	30.0	35.0	./.	./.
**7**	Ekroth [30]	2012	12.0	12.0	12.0	./.	./.	./.	7.0	60.7	./.	./.
**8**	Herzberg [61]	2012	112.0	112.0	./.	./.	./.	./.	112.0	20.5	./.	./.
**9**	Morapudi [82]	2012	21.0	21.0	21.0	55.1	22.5	87.0	21.0	44.8	4.3	83.3
**10**	Nydick [4]	2012	23.0	23.0	23.0	./.	./.	./.	23.0	31.0	./.	./.
**11**	Reigstad [45]	2012	27.0	27.0	30.0	43.0	./.	./.	27.0	19.2	./.	./.
**12**	Pierrat [17]	2012	11.0	11.0	11.0	61.6	./.	./.	11.0	42.9	./.	./.
**13**	Boeckstyns [58]	2013	65.0	52.0	52.0	58.0	14.0	89.0	28.0	42.0	0.0	84.0
**14**	Marcuzzi [21]	2014	35.0	35.0	35.0	56.9	16.7	95.0	35.0	11.4	1.0	50.8
**15**	Badge [52]	2016	85.0	85.0	40.0	61.3	16.0	91.0	59.0	45.8	0.0	89.0
**16**	Chevrollier [63]	2016	17.0	15.0	15.0	./.	./.	./.	15.0	29.0	2.3	65.9
**17**	Reigstad [46]	2017	37.0	37.0	48.0	38.0	./.	./.	48.0	25.0	./.	./.
**18**	Honecker [62]	2017	23.0	23.0	23.0	57.9	./.	./.	23.0	37.9	./.	./.
**19**	Giacalone [20]	2017	25.0	25.0	25.0	./.	./.	./.	25.0	20.0	./.	./.
**20**	Giwa [44]	2018	25.0	25.0	25.0	57.6	./.	./.	25.0	21.1	./.	./.
**21**	Kennedy [54]	2018	48.0	48.0	48.0	58.2	./.	./.	48.0	25.4	./.	./.
**22**	Bellemere [18]	2019	51.0	51.0	51.0	63.0	./.	./.	51.0	34.0	./.	./.
**23**	Friedel [83]	2019	9.0	9.0	9.0	./.	./.	./.	9.0	48.0	./.	./.
**24**	Froschauer [60]	2019	39.0	39.0	39.0	63.0	./.	./.	39.0	29.0	./.	./.
**25**	Matsui [77]	2019	20.0	20.0	20.0	61.2	./.	./.	20.0	36.1	./.	./.
**26**	Zijlker [56]	2019	26.0	26.0	26.0	./.	./.	./.	26.0	41.0	./.	./.
**27**	Biehl [70]	2021	34.0	34.0	34.0	./.	./.	./.	34.0	47.1	1.7	88.8
**28**	Lestienne [16]	2021	28.0	28.0	28.0	62.0	34.0	100.0	28.0	36.0	0.0	75.0

**Table 7:** Grip strength (./.: Data are not available within a study; n is the number of included procedures/prosthesis).

No.	Reference	Year of Publication	Number of Procedures Included	Number Included for Follow Up	Grip Strength (kg)							
			(n)	(n)	Preoperatively (n)	Mean	Range from	Range to	Postoperatively (n)	Mean	Range from	Range to
**1**	**Meuli** [48]	1995	49.0	49.0	10.0	15.0	10.0	25.0	10.0	25.0	10.0	25.0
**2**	Levadoux [47]	2003	28.0	28.0	28.0	20.0	5.0	35.0	28.0	32.0	10.0	70.0
**3**	Rizzo [39]	2003	17.0	17.0	17.0	5.6	./.	./.	17.0	9.8	./.	./.
**4**	Bidawi [57]	2012	10.0	10.0	10.0	2.1	./.	./.	10.0	7.9	./.	./.
**5**	Cooney [59]	2012	46.0	30.0	30.0	10.0	./.	./.	30.0	13.0	./.	./.
**6**	Herzberg [61]	2012	112.0	112.0	112.0	./.	./.	./.	112.0	29.5	./.	./.
**7**	Pierrart [17]	2012	11.0	11.0	11.0	20.4	./.	./.	11.0	8.3	./.	./.
**8**	Reigstad [45]	2012	27.0	27.0	30.0	22.6	./.	./.	27.0	22.8	./.	./.
**9**	Boeckstyns et al.	2013	65.0	52.0	52.0	10.0	./.	./.	52.0	15.0	./.	./.
**10**	Marcuzzi [21]	2014	35.0	35.0	35.0	10.1	2.0	29.3	35.0	16.5	2.6	42.8
**11**	Badge [52]	2016	85.0	85.0	46.0	4.8	1.7	11.5	37.0	10.2	0.0	28.0
**12**	Chevrollier [63]	2016	17.0	15.0	15.0		./.	./.	15.0	17.3	8.0	27.0
**13**	Reigstad [46]	2017	37.0	37.0	48.0	21.0	./.	./.	48.0	24.0	./.	./.
**14**	Honecker [62]	2017	23.0	23.0	23.0	7.6	./.	./.	23.0	13.9	./.	./.
**15**	Giwa [44]	2018	25.0	25.0	25.0	12.3	./.	./.	25.0	27.8	./.	./.
**16**	Lestienne [16]	2021	28.0	28.0	28.0	10.0	4.0	23.0	28.0	17.0	8.0	27.0

**Table 8:** Range of Motion (RoM)—the table showed the RoM preoperatively compared to the postoperative situation. The separation between flexion and extension and radial and ulnar deviation leads to the overall motion (./.: Data are not available within a study).

No.	Reference	Year of Publication	Preoperatively						Postoperatively						Additional Information
			Flexion	Extension	Overall FE	Radial	Ulnar	Overall RUD	Flexion	Extension	Overall FE	Radial	Ulnar	Overall RUD	
**1**	**Figgie** [74]	**1983**	./.	./.	35.0	./.	./.	./.	./.	./.	50.0	10.0	10.0	20.0	
**2**	Bosco [71]	1994	./.	./.	./.	./.	./.	./.	17.0	32.0	49.0	2.0	23.0	25.0	Active Range of Motion
**3**	Meuli [48]	1995	./.	./.	./.	./.	./.	./.	30.0	40.0	70.0	10.0	10.0	20.0	
**4**	Cobb [36]	1996	34.0	23.0	57.0	5.0	16.0	21.0	29.0	36.0	65.0	10.0	20.0	30.0	Last follow up
**5**	Gellman [72]	1997	9.6	13.9	23.5	3.2	5.0	8.2	10.3	18.2	28.5	7.8	13.2	21.0	
**6**	Menon [66]	1998	20.0	37.0	57.0	4.0	12.0	16.0	36.0	41.0	77.0	7.0	13.0	20.0	
**7**	Courtman [37]	1999	./.	./.	50.0	./.	./.	17.0	./.	./.	36.0	./.	./.	32.0	
**8**	Divelbiss [64]	2002	./.	./.	./.	./.	./.	./.	41.0	35.0	76.0	9.0	19.0	28.0	after 2 years
**9**	Takwale [42]	2002	./.	./.	./.	./.	./.	./.	28.8	17.4	46.2	6.0	13.6	19.6	
**10**	Levadoux [47]	2003	26.0	20.0	46.0	7.0	25.0	32.0	48.0	41.0	89.0	12.0	22.0	34.0	
**11**	Radmer [50]	2003	./.	./.	./.	./.	./.	./.	35.0	34.0	69.0	7.0	17.0	24.0	
**12**	Rahimtoola [51]	2003	26.0	7.0	33.0	2.0	10.0	12.0	35.0	24.0	59.0	10.0	15.0	25.0	
**13**	Rizzo [39]	2003	20.0	29.0	49.0	4.0	22.0	26.0	23.0	34.0	57.0	9.0	25.0	34.0	
**14**	Rahimtoola [69]	2004	23.0	23.0	46.0	6.0	11.0	17.0	32.0	31.0	63.0	8.0	16.0	24.0	
**15**	Stegeman [40]	2005	17.0	17.0	34.0	3.0	6.0	9.0	41.0	41.0	82.0	14.0	31.0	45.0	
**16**	Kistler [75]	2005	./.	./.	./.	./.	./.	./.	28.0	15.0	43.0	7.0	14.0	21.0	
**17**	Kretschmer [38]	2007	29.0	31.0	60.0	12.0	18.0	30.0	32.0	36.0	68.0	13.0	20.0	33.0	
**18**	Strunk [41]	2009	./.	./.	./.	./.	./.	./.	25.6	24.5	50.1	8.0	13.0	21.0	
**19**	van Winterswijk [67]	2010	21.0	30.0	51.0	5.0	12.0	17.0	29.0	38.0	67.0	7.0	17.0	24.0	
**20**	Ferreres [53]	2011	./.	./.	./.	./.	./.	./.	42.0	26.0	68.0	1.0	26.0	27.0	
**21**	Ward [68]	2011	32.0	16.0	48.0	6.0	15.0	21.0	42.0	20.0	62.0	8.0	17.0	25.0	
**22**	van Harlingen [43]	2011	21.0	18.0	39.0	5.0	4.0	9.0	29.0	28.0	57.0	10.0	19.0	29.0	
**23**	Bidawi [57]	2012	./.	./.	./.	./.	./.	./.	22.5	34.5	57.0	6.8	15.5	22.3	
**24**	Cooney [59]	2012	./.	./.	./.	./.	./.	./.	30.0	38.0	68.0	8.0	20.0	28.0	
**25**	Ekroth [30]	2012	./.	./.	./.	./.	./.	./.	./.	./.	54.5	./.	./.	28.0	
**26**	Herzberg [61]	2012	./.	./.	./.	./.	./.	./.	33.0	23.5	65.6	7.5	26.0	33.5	mean Non RA and RA
**27**	Morapudi [82]	2012	16.7	20.9	37.6	./.	./.	./.	22.4	30.5	52.9	./.	./.	./.	
**28**	Nydick [4]	2012	45.0	40.0	85.0	8.0	27.0	35.0	43.0	47.0	90.0	14.0	29.0	43.0	
**29**	Pierrart [17]	2012	44.1	34.5	78.6	13.7	17.5	31.2	35.0	36.5	71.5	10.0	25.6	35.6	Last follow up
**30**	Reigstad [45]	2012	./.	./.	104.0	./.	./.	./.	./.	./.	120.0	./.	./.	./.	after 1 year
**31**	Boeckstyns [58]	2013	31.0	30.0	61.0	8.0	16.0	24.0	31.0	29.0	60.0	6.0	22.0	28.0	all cases
**32**	Marcuzzi [21]	2014	25.0	25.0	50.0	4.7	12.0	16.7	33.0	34.0	67.0	5.3	19.0	24.3	
**33**	Badge [52]	2016	19.1	20.8	39.9	6.1	14.7	20.8	29.1	30.7	59.8	4.0	14.2	18.2	
**34**	Chevrollier [63]	2016	./.	./.	./.	./.	./.	./	./.	./.	33.0	./.	./.	20.0	
**35**	Gil [65]	2017	./.	./.	./.	./.	./.	./.	37.0	29.0	66.0	./.	./.	./.	
**36**	Honecker [62]	2017	35.4	34.3	69.7	./.	./.	./.	38.7	44.7	83.4	./.	./.	./.	
**37**	Pfanner [55]	2017	./.	./.	./.	./.	./.	./.	./.	./.	53.4	./.	./.	18.4	mean of all cases
**38**	Giacalone [20]	2017	./.	./.	./.	./.	./.	./.	27.0	33.0	60.0	12.0	27.0	39.0	
**39**	Giwa [44]	2018	./.	./.	78.4	./.	./.	35.2	./.	./.	112.3	./.	./.	40.4	
**40**	Kennedy [54]	2018	./.	./.	./.	./.	./.	./.	33.0	24.0	57.0	./.	./.	./.	
**41**	Bellemere [18]	2019	./.	./.	66.0	./.	./.	./.		./.	75.0	./.	./.	./.	
**42**	Friedel [83]	2019	./.	./.	./.	./.	./.	./.	31.0	29.0	60.0	./.	./.	./.	
**43**	Froschauer [60]	2019	20.0	20.0	40.0	5.0	15.0	20.0	40.0	35.0	75.0	15.0	30.0	45.0	
**44**	Matsui [77]	2019	./.	./.	42.3	./.	./.	./.	./.	./.	48.2	./.	./.	./.	Last follow up
**45**	Biehl [70]	2021	26.8	20.8	47.6	12.0	16.9	28.9	26.5	12.3	38.8	25.3	9.2	34.5	
**46**	Lestienne [16]	2021	./.	./.	./.	./.	./.	./.	33.0	33.0	66.0	10.0	20.0	30.0	Last follow up

**Table 9:** Pronation and Supination of the forearm (./.: Data are not available within a study).

No.	Reference	Year of Publication	Preoperatively			Postoperatively		
			Pronation	Supination	Overall	Pronation	Supination	Overall
**1**	**Cobb** [36]	1996	69.0	65.0	134.0	73.0	67.0	140.0
**2**	Divelbiss [64]	2002	./.	./.	./.	88.0	80.0	168.0
**3**	Levadoux [47]	2003	60.0	45.0	105.0	90.0	77.0	167.0
**4**	Rahimtoola [51]	2003	77.0	46.0	123.0	83.0	57.0	140.0
**5**	Rizzo [39]	2003	68.0	61.0	129.0	75.0	66.0	141.0
**6**	Rahimtoola [69]	2004	73.0	66.0	139.0	88.0	82.0	170.0
**7**	Strunk [41]	2008	./.	./.	./.	82.0	71.0	153.0
**8**	Ward [68]	2011	54.0	50.0	104.0	83.0	71.0	154.0
**9**	van Harlingen [43]	2011	80.0	70.0	150.0	85.0	90.0	175.0
**10**	Cooney [59]	2012	./.	./.	./.	75.0	70.0	145.0
**11**	Pierrart [17]	2012	81.5	72.5	154.0	83.5	88.0	171.5
**12**	Reigstad [45]	2012	87.0	83.0	170.0	82.0	85.0	167.0
**13**	Boeckstyns [58]	2013	79.0	71.0	150.0	81.0	83.0	164.0
**14**	Reigstad [46]	2017	82.0	81.0	163.0	83.0	83.0	166.0
**15**	Honecker [62]	2017	72.3	68.3	140.6	75.1	77.8	152.9
**16**	Giwa [44]	2018	./.	./.	136.7	./.	./.	137.2
**17**	Biehl [70]	2021	60.0	65.0	125.0	58.4	79.0	137.4
**18**	Lestienne [16]	2021	66.0	64.0	130.0	73.0	75.0	148.0
